# A Snapshot of T Cell Subset Cytokines in Pemphigus Vulgaris: A Cross-Sectional Study

**DOI:** 10.7759/cureus.29890

**Published:** 2022-10-04

**Authors:** Praveen K Singh, Shukla Das, Gargi Rai, Mohammad A Ansari, Sajad A Dar, Taru Singh, Deepika Pandhi

**Affiliations:** 1 Microbiology, University College of Medical Sciences, New Delhi, IND; 2 College of Nursing and Allied Health Sciences, Jazan University, Jazan, SAU; 3 Dermatology, University College of Medical Sciences and Guru Teg Bahadur Hospital, New Delhi, IND

**Keywords:** treg, th17, th1, cytokine, pemphigus vulgaris

## Abstract

Objective: The purpose of this study was to assess the serum levels of cytokines produced by the Th1 (IFN‐γ, IL-12), Th2 (IL‐4), Th17 (IL-6, IL‐17A, IL‐23), and Treg (IL‐10 and TGF-β) pathways in individuals with active pemphigus vulgaris (PV) and to determine whether these levels were correlated with the severity of the disease condition.

Patients and methods: This study was conducted with 90 individuals, of which 50 were PV patients and 40 healthy individuals (age and gender-matched) as controls. Serum samples were collected and tested for cytokine levels by ELISA (enzyme-linked immunosorbent assay). The cytokine levels in the serum of PV patients and healthy controls were compared statistically using the Mann-Whitney test for nonparametric samples. The strength of the association between the variables was evaluated using the Spearman correlation test.

Results: The mean serum levels of IFN- γ (p < 0.001), IL-6 (p < 0.001), IL-10 (p < 0.001), IL-12 (p < 0.05), and IL-17 (p < 0.001) were significantly higher and TGF-β were significantly low in the PV patients than those observed in the control group. The mean concentration of serum IL-4 in patients with PV did not differ from those in the control group.

Conclusions: In active PV, the Th1 and Th17 pathways are involved in the development and progression of the disease, whereas the Th2 pathway is blocked. Both of these pathways play a significant role in the disease. It is possible that the Treg pathway acts as an antagonist to the Th1 and Th17 pathways, which would cause the disease to become more localised. This study lays the foundation for a better understanding of the aetiology of PV and implies that cytokines could be used as potential therapeutic targets and disease activity biomarkers.

## Introduction

Pemphigus vulgaris (PV) is a life-threatening autoimmune blistering disease resulting from the formation of IgG autoantibodies directed against autoantigen desmoglein 3 (Dsg3) within the epidermis and mucous membranes. Oral and skin manifestations are common with flaccid epidermal blisters and acantholysis [1]. PV affects 0.1-3.2/100,000 individuals per year with substantial morbidity and increased mortality [2,3]. Although the aetiology of PV is unknown, the genetic component, environmental factors, infections, and hormonal factors seem to have a significant impact on triggering the autoimmune response and driving the pathogenesis of the disease.

Heightened and renegade immune responses lead to the initiation and progression of PV through myriad mechanisms. This activity is manifested by changes in the synthesis of some cytokines, activation of the complement system, and unchecked B lymphocyte hyperactivity. This disrupts cellular and immunological homeostasis and further orchestrates immunoglobulin class changes, leading to an increase in the production of autoantibodies and a breakdown in cell-mediated immunity that shows up as abnormalities in T lymphocytes and antigen-presenting cells (APCs) [4].

The population of CD4+ T cells with different subsets secrete restricted and non-overlapping sets of cytokines from other subsets and mediate responses in pathological conditions. Early after activation, naïve T cells can produce multiple cytokines, but progressive activation and repeated stimulation lead to polarisation and the production of selected cytokines. The effector CD4 + Th lymphocytes and their characteristic cytokines can be grouped into Th1 (interferon (IFN- γ, IL-12, and IL-2), Th2 (IL-4, IL-5, IL-6, IL-10, IL-13), and Th17 (IL-6, IL-17, IL-22, and IL-23) subsets based on cytokine secretion profile [5-7]. However, the production of IL-2, IL-10, and IL-6 is not restricted to a single subset; thus, TH1/TH2 subsets are often characterised by IFN-γ/IL-4 secretion [5,7-9]. Previous studies documented that extrinsic IFN-γ and IL-12 cytokine signals are needed to drive the differentiation of Th1 cells from naïve CD4+ T cells by the expression of transcriptional regulator T-bet and production of IFN-γ. The IFN-γ by autocrine feedback loop further enhances the polarisation of Th1 cells. Th2 cells required extrinsic IL-4 and expressed the transcriptional factor GATA3, which resulted in the production of cytokines such as IL-4, IL-5, and IL-13 [10].

However, apart from TH1 and TH2 lymphocytes, TGF-β and IL-1, IL-6, or IL-23 cytokine signals also caused naive CD4+ T-cell differentiation into TH17 cell subsets. TH17 cells are in turn characterised by the expression of the transcription factor (related orphan receptor gamma; RORγt) and cause the production of IL-17 and IL-22 cytokines [11,12]. Recent studies indicated that Th17 cells were increased and their associated molecules (IL-17A and CCL20) in the serum were elevated in PV patients. These findings shed light on the importance of Th17 cells and IL-17 in the development of PV [13].

Another subset of CD4+ T cells is T-regulatory (Tregs) cells, which are capable of suppressing autoreactive effector T cells and controlling most immune responses through several mechanisms. A decrease in the number of these cells, as well as their dysfunction, may lead to the development of autoimmune diseases [14]. Although the hypothesis of Treg cells playing a preventative role in autoimmunity is well acknowledged, findings on PV are conflicting [13,15].

There is still debate, and further research is needed to establish the significance of certain T cell subsets and related cytokines in the pathogenesis of PV. In this study, we propose that evaluating the circulating cytokine milieu may shed light on the postulated Th cell imbalance mechanism in PV. In order to achieve this aim, we conducted this study to assess the serum levels of T cell subsets' signature cytokines.

## Materials and methods

Study population

A total of 50 consecutive PV patients (both sexes) undergoing treatment for the active disease were enrolled in the present study. Disease activity was measured by the Pemphigus Disease Area Index (PDAI) from the patients on the days of blood sampling. Patients having any history of active/chronic infection or disease other than PV, on treatment in the form of topical or systemic steroids, antibiotics, or any long-term therapy in the preceding six months of presentation, nursing mothers, pregnant women, and elderly patients were excluded from this study. Forty age- and sex-matched healthy volunteers selected on the basis of the absence of any history or evidence of PV or any other active disease were also enrolled.

All investigations were done in accordance with the University College of Medical Sciences (University of Delhi) and Guru Teg Bahadur Hospital, Health and Human Ethical Clearance Committee. After collecting peripheral blood, the samples were allowed to stand for half an hour and then centrifuged (Remi R-8C centrifuge) at 3000 revolutions per minute for five minutes. Following the separating of the serum samples and the aliquoting of them, they were stored in the freezer at a temperature of −80 °C for further cytokine analysis. All of the PV patients and healthy controls gave their written informed consent to participate in the study.

Laboratory and immunological investigations

When the blood samples were collected from all of the patients and the healthy controls, detailed medical histories were documented. Only patients with a confirmed clinical diagnosis of PV by histopathology, direct immunofluorescence microscopy, and the detection of circulating autoantibodies by indirect immunofluorescence microscopy (intercellular IgG binding to epithelial cells) and/or by a commercial Dsg3-ELISA were enrolled. Pemphigus vulgaris was considered to be active when patients had blisters or sores on their skin or mucous membranes.

Cytokine assay

The levels of circulating cytokines in the serum of patients and controls were determined by commercial Diaclone ELISA kits of IL-4(0.7 pg/ml), IL-6 (2 pg/ml), IL-10(4.9 pg/ml), IL-12(2.2 pg/ml), IL-17 (2.3 pg/ml), IL-23 (20 pg/ml), IFN-γ (0.69 pg/ml), and TGF- β (8.6 pg/ml) as per the manufacturer's guidelines. Using a microplate reader, we determined the absorbance of each well at 450 nm. Each serum sample was analyzed twice to ensure accuracy. The digital data of raw absorbency value were easily processed by the ELISA reader-controlling software into a standard curve from which the cytokine levels of patients and healthy volunteers were directly determined.

Statistical analysis

The GraphPad Prism (version 8, GraphPad Software, La Jolla, CA, USA) was used to analyze the data. Continuous variables were summarized through the mean ± standard deviation (SD). The difference in the averages between the study groups was examined by the Mann-Whitney U test followed by Spearman correlation to evaluate the strength of association between the variables. All of the results were considered significant at p<0.05.

## Results

Patient profile

The demographic and clinical characteristics of PV patients are summarised in Table 1.

**Table 1:** Demographic and clinical characteristics of patients with pemphigus vulgaris. PV: pemphigus vulgaris, PDAI: pemphigus disease area index.

Parameter	PV patients (N=50)
Gender (male/female)	18:32
Age mean (range) years	41.51 ± 12.06
Duration of disease (years, mean ±SD)	1.43 ± 1.71
Clinical features (n)(%)	Mucocutaneous disease n=46 (92%), mucosal n=3 (6%), cutaneous n=1(2%)
Disease activity index (PDAI score)	25.44±21.33
Dsg1	61.18
Dsg3	160.23±72.38

Cytokines secretion profile in relation to PV diseases

**Figure 1:**
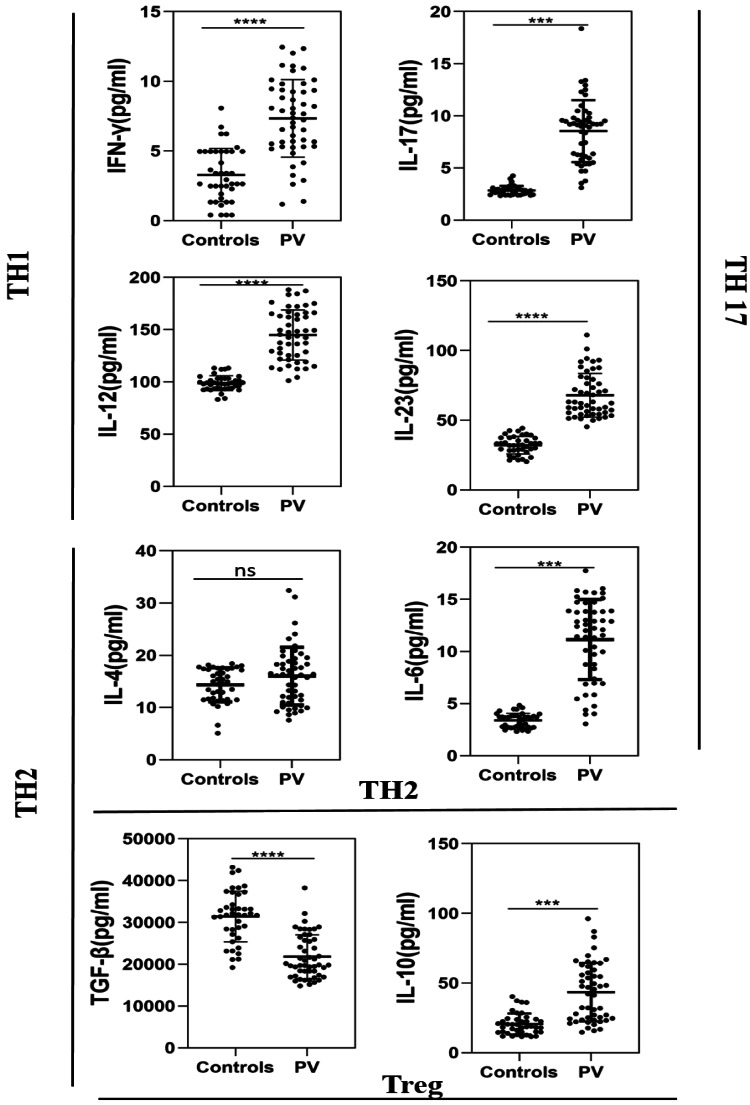
Levels of IL-6, IL-17, IL-23, IL-4, IL-10, IFN-γ, TGF-β, and IL-12 in the blood sample of patients and healthy controls. The Mann–Whitney test was utilised, and the findings are presented as the mean ± standard error. TGF-β: transforming growth factor and IFN-γ: Interferon gamma. Groups statistically significantly different from controls; *p < 0.05, **p < 0.01; ***p < 0.001, ****p < 0.0001, ns=non-significant.

In this study, we investigated the relationship between cytokine secretion profiles and PV illnesses. Figure 1 demonstrates that the levels of IFN-γ (p <0.0001) IL-6 (p< 0.001), IL-10 (p < 0.001), IL-12 (p < 0.0001), IL-17 (p < 0.0001), and IL-23(p < 0.0001) were all significantly greater in PV patients than in controls, although the levels of IL-4 were comparable in both groups. Patients diagnosed with PV have TGF-β (p < 0.0001) levels significantly lower than those of the control group (Table 2).

**Table 2:** The serum level of various cytokines in patients and controls.

Cytokines level (pg/mL)	Mean ± SD		P-value
	Patient (pg/mL) (n = 50)	Control (pg/mL) (n = 40)	
IFN‐γ	7.345±2.790	3.277±1.914	<0.0001
IL‐4	16.03±5.531	14.36±3.269	0.2363
IL-6	11.15±3.839	3.388±0.6955	<0.001
IL-10	43.42±20.96	20.56±7.563	<0.001
IL-12	144.9±24.06	98.82±7.078	<0.0001
IL-17	8.538±2.975	2.838±0.4330	<0.001
IL-23	32.02±6.373	8.538±2.975	<0.0001
TGF-β	21807±5269	31434±6047	<0.0001

**Figure 2:**
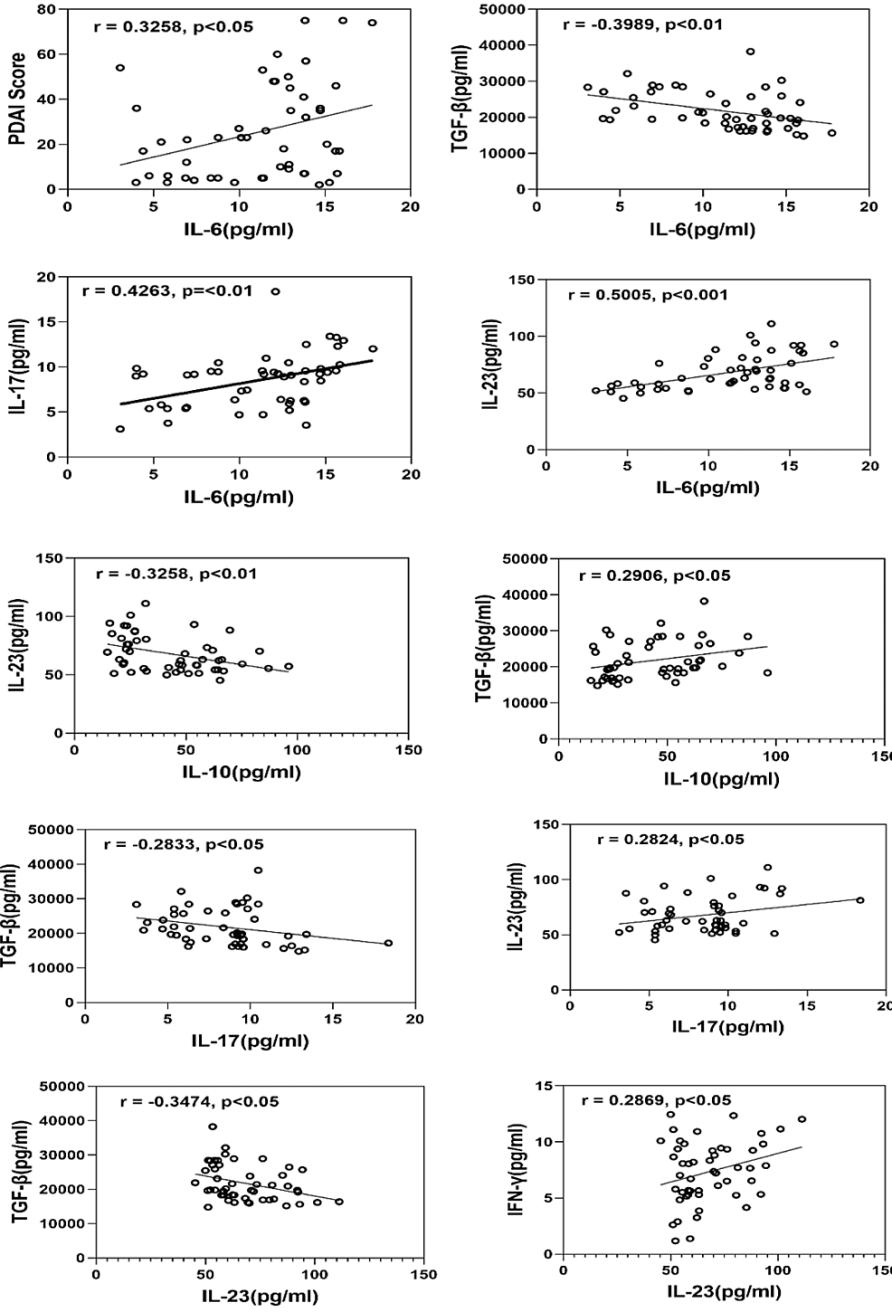
The relationship between various cytokines and the PDAI score in PV patients. To assess the strength of correlation between the variables, the Spearman correlation test was performed.

Regarding the correlation of cytokines among PV patients, IL-6 was found to be positively correlated with IL-17 (r = 0.4263, p ≤ 0.01) and IL-23 (r = 0.5005, p < 0.001), but negative correlated with TGF-β (r = −0.3989, p<0.01) (Figure 2). Although there was a positive correlation between IL-10 and TGF-β (r = 0.2906, p<0.05), it was negatively correlated with IL-23 (r = −0.3258, p < 0.01). Both IL-17 (r = -0.2833, p<0.05) and IL-23 (r = −0.3474, p < 0.05) were found to have a negative correlation with TGF-β, respectively. It was observed that IL-23 has a positive association with both IL-17 (r = 0.2824, p<0.05) and IFN- γ (r = 0.2869, p<0.05).

There was no statistically significant correlation found between the variable cytokines in this investigation and the drugs taken by PV patients (p > 0.05; data not shown). To assess the relationship between cytokines and disease activity, PV patients were categorised into mild, moderate, and severe disease groups based on the PDAI. We discovered no significant difference between these groups in terms of cytokine concentration. As shown in Figure 2, we found a strong correlation between IL-6 and PDAI scores in individuals with PV (r = 0.3258, p < 0.05).

## Discussion

Cytokines are the key to the immune system's communication cascade. To unravel the predisposition of PV disease and its mechanism, unveiling the role of cytokines becomes indispensable. However, the literature available regarding PV is obscure and ambiguous, which does not allow a concept to get metamorphosed. Hence, this study attempts to plug the current loopholes through the study of T cell subsets' cytokine secretion characterization in PV patients.

In this study, we found that the serum levels of IFN-γ in patients are considerably higher than in healthy controls. Our findings are consistent with the majority of the studies that demonstrate higher IFN-γ levels in patients [5,7,8]. Increased serum IFN-γ levels are indicative of the previously documented decrease in the frequency of Th1 cells in PV. IL-4 is a cytokine that is characteristically generated by Th2 cells. The current study also showed that there is no statistically significant change in the serum level of the Th2 cytokine IL-4 in PV. The findings of Lee et al., who likewise showed no change in IL-4 serum levels in the PV patients, lend credence to this conclusion [7]. However, a few studies have found a substantial rise in IL-4 blood levels in patients with PV compared to healthy controls [5,9,16].

IL-10 is essential to the diagnosis of PV. IL-10 levels are frequently shown to be elevated in patients with PV, making them a helpful marker for PV characterisation [5,9,17]. The aberrant proliferation of B cells and monocytes in PV is accompanied by markedly elevated IL-10 levels [18]. We were able to show a considerably higher level of IL-10 in individuals with PV, which is consistent with earlier research. In addition, our study sheds insight into the function of IL-6, IL-17, and IL-23. A normal immune response requires a delicate equilibrium of these cytokines. We observed elevated amounts of IL-6 and IL-17, which together promote the formation of Th17 cells. These data demonstrate that the delicate balance between IL-6 and IL-17 is disrupted in PV, resulting in an elevated IL-17 population in CD4+ T cells. According to numerous studies, higher levels of IL-6 indicate B-cell hyperactivity, autoantibody production, and additional immunopathology of PV [9,17,19,20].

Moreover, earlier investigations have revealed increased IL-17 levels in patients with PV [9,17,21]. Increased IL-17-producing cells led to increased IL-17 levels. The ratio of Th1 to Th17-related blood cytokine levels was higher in PV patients compared to healthy controls. This Th17 to Th1 cell axis provides evidence that patients with PV have an abnormal CD4+ T cell response. This intensified CD4+ T-cell response increases the frequency of Th17 cells. Among the cytokines implicated in Th-17 proliferation is IL-23. The proliferation of Th17 cells is linked to a paradigm involving IL-6 and IL-23 [22]. The pathogenesis of numerous autoimmune illnesses is caused by an unequal distribution of the IL-12-unique P35 subunit and the IL-12 and IL-23-shared P40 subunit. Numerous studies have highlighted the involvement of IL-23 in the advancement of organ-specific autoimmune illness through the stimulation of Th17 cells, which dominate the target organ, leading to the onset of inflammation, confirming the relationship between IL-6 and IL-23. In addition, we have investigated IL-12 in individuals with PV. Based on our findings, IL-12 levels were increased in cases compared to controls, which is consistent with prior research [20]. As the primary cells that produce IL-12, dendritic cells (DC) play a vital part in PV, and this provides information regarding their function [23]. The regulation of T-cell responses is highly dependent on IL-12 family cytokines (IL-12, IL-23, and IL-27). The expression of these responses is the result of a signalling cascade involving monocytes, macrophages, and DC cells, which are recruited in response to an infection and generate cytokines belonging to the IL-12 family [24].

IL-6 and IL-17 levels were shown to be substantially higher in PV. Based on the observation that higher amounts of IL-17 are found in PV, we postulate that Th17 cells play a significant role. This shows that the frequency of circulating Il-17-producing TH17 cells and the propensity to produce IL-17 were elevated in patients with PV [25].

In addition, our study demonstrates that TGF-β1 levels in PV were lower than in the control group. Earlier studies have shown that TGF-β1 levels in the blood of PV patients are lower, which is related to fewer peripheral Treg cells [21,26]. Th17 cells and induced Treg cells require TGF-β1 in the presence of pro-inflammatory cytokines such as IL-6 during the naive stage of development [27]. This gives us a hint as to why a decrease in TGF-β in lymphatic tissue may initiate immunological dysregulation and autoantibody formation. FoxP3, a crucial transcription factor for the peripheral Treg subgroup, is activated by TGF-, which is important for peripheral Treg division. FoxP3+ Treg activation is impaired in the presence of pro-inflammatory cytokines such as IL-6, which promote the formation of Th17 cells. While we had previously seen an increase in serum IL-10 levels, the current pattern of decreased TGF-β1 and elevated IL-6 and IL-17 is more indicative of Th17 activity than Treg activity. The autoimmune condition may emanate when CD4+ T cell development is lopsided against Treg cells towards the Th17 cell phenotype [28]. The deficiency of Treg cells and reduced Treg/Th17 ratio are characteristic of PV patients in diseased conditions [29]. A further deficiency of IFN-γ(Th1) and TGF-β is correlated with enhanced IL-6 and IL-17(Th17), which supports Timoteo et al. that in PV there is a relative dearth of Th1 and Treg cells as compared to th17 cell subsets [30]. This asymmetry is the hallmark of PV because patients with quiescent disease display a Th17/Treg ratio that favours Th17 cells.

Although an immunosuppressive treatment regimen is used to cure PV, these immunosuppressive treatments do not correlate fully with variable cytokines in PV patients [9]. This weak correlation may be the result of high PDAI variability and heterogeneity in the types of PV involvement.

PV is one of the most chronic autoimmune disorders and is defined by an imbalance in T-cell responses (Th1, Th2, Th17, and Treg cells) in peripheral blood. Most of the cytokines we tested, including IFN-γ, IL-6, IL-10, IL-12, IL-17, IL-23, and TGF-β, exhibited statistically significant variations. Prior studies have attempted to enlighten our understanding of the role of Th17 as a predisposing factor in the inflammatory process of PV [13,19]. Our data are in agreement with and corroborate these reports. In addition, this Th17 aggravation is accompanied by a decrease in peripheral blood Treg cells. Numerous studies have demonstrated that blood levels of IL-6 and IL-17 are elevated and that this elevated serum level correlates with severe disease activity [19,20,30].

Study limitation

In this study, at this juncture, we would like to mention that more lucid information is awaited due to the small sample size. It is possible that a more extensive study that includes a longitudinal evaluation of these cytokines will reveal their connection to the activity of the disease and the therapeutic response.

## Conclusions

In this study, we report a rise in serum concentration of IFN-γ, IL-6, IL-10, IL-12, IL-17, IL-23 cytokine and a decrease in TGF-β concentration in PV patients. These findings would aid the scientific community in elucidating Th1 association with Th17 cells as a predisposing factor for the advent of PV. Although there were minimal reports addressing the significance of Th17 in the development of PV, there were insufficient investigations to demystify and clarify Th17's function. Using suitable statistical comparison and a cross-sectional methodology, we were able to report the asymmetric balance of Th1/Th17 and Treg/Th17 (based on serum cytokine profile) in PV patients in our study. In addition, we propose that further functional research is required to comprehend the TH1/TH17/Treg imbalance in peripheral blood, particularly during the active phase of the disease, which will aid in the development of therapeutic approaches.
